# Therapeutic potential of brentuximab vedotin in breast cancer and lymphoma via targeted apoptosis and gene regulation

**DOI:** 10.1038/s41598-024-84744-y

**Published:** 2025-01-13

**Authors:** Abeer Ezzat, Mohga Shafiek, Shimaa Shawki, Shaimaa Abdel-Ghany, Mahmoud Nazih, Hussein Sabit

**Affiliations:** 1 Department of Medical Biotechnology, College of Biotechnology, Misr University for Science and Technology, P. O. Box 77, Giza, Egypt; 2 Department of Chemistry, Faculty of Science, Helwan University, Cairo, 11795 Egypt; 3 Department of Environmental Biotechnology, College of Biotechnology, Misr University for Science and Technology, P. O. Box 77, Giza, Egypt; 4 Scientific Office, Egyptian Society of Pharmacogenomics and Personalized Medicine (ESPM), Cairo, Egypt; 5 Department of Clinical Pharmacy, Faculty of Pharmacy, Ahram Canadian University (ACU), 6Th of October City, Giza 12566 Egypt

**Keywords:** Brentuximab, Breast cancer, Apoptosis, Cell cycle analysis, Gene expression, Breast cancer, Immunology

## Abstract

This study was designed to assess the effect of brentuximab vedotin on several breast cancer cell lines in terms of promoting apoptosis and managing cancer progression. Additionally, the study investigated the potential of repurposing this drug for new therapeutic reasons, beyond its original indications. The study evaluates the cytotoxic effects of Brentuximab vedotin across five cell lines: normal human skin fibroblasts (HSF), three breast cancer cell lines (MCF-7, MDA-MB-231, and T-47D), and histiocytic lymphoma (U-937). Brentuximab treatment was administered at four time points (0, 24, 48, and 72 h), with cell viability assessed at each interval. HSF cells, serving as controls, exhibited minimal viability loss (above 70%), indicating limited toxicity in normal fibroblasts. In contrast, MCF-7 and MDA-MB-231 cells demonstrated time-dependent reductions in viability, with a pronounced decline by 72 h, suggesting Brentuximab’s efficacy in both ER-positive and triple-negative breast cancer. T-47D cells also showed decreased viability, though at a slower rate. U-937 cells exhibited the most substantial reduction, highlighting Brentuximab’s potent activity against hematologic malignancies. Wound healing assays further revealed that Brentuximab significantly impaired the migration and healing capacity of cancer cells compared to untreated controls. Additionally, cell cycle analysis indicated G2/M phase arrest in cancer cells, particularly in MCF-7 and MDA-MB-231, while HSF cells remained largely unaffected. Apoptosis detection confirmed Brentuximab-induced cell death, with significant increases in late apoptosis in cancer lines, especially by 72 h. Gene expression analysis revealed upregulation of pro-apoptotic genes (BAX, Caspase 3, and Caspase 9) in cancer cells, alongside a decrease in anti-apoptotic BCL-2 expression. These findings suggest Brentuximab’s selective cytotoxicity against cancer cells and its potential as an effective therapeutic agent, particularly in breast cancer and histiocytic lymphoma.

## Introduction

Breast cancer ranks as the second most prevalent form of cancer globally, representing approximately 11.6% of all cancer cases, as reported by the World Health Organization^1^. It is responsible for about 25% of all cancer diagnoses and 16% of cancer-related deaths among women worldwide^2^. Projections suggest that by 2040, the number of new breast cancer cases will surpass three million annually^3^. Although breast cancer is often categorized as a singular disease, its management presents considerable challenges due to the existence of multiple subtypes, each requiring distinct therapeutic strategies. The majority of women diagnosed with breast cancer will undergo surgery to remove the tumor^4^.

A promising approach to advancing cancer therapy involves developing treatments that exploit the differential expression of specific molecules between cancerous and healthy cells. This strategy aims to enhance the selectivity of treatments, targeting tumor cells while reducing collateral damage to normal tissues. One prominent class of such therapies is antibody–drug conjugates (ADCs). These cutting-edge treatments combine the high specificity of monoclonal antibodies (MAbs) with potent cytotoxic agents, enabling the selective delivery of these drugs to cells expressing particular antigens. The clinical success of ADCs, demonstrated by agents like brentuximab vedotin in hematologic malignancies and ado-trastuzumab emtansine in solid tumors, underscores their substantial therapeutic potential^5^.

The treatment strategy for breast cancer is largely determined by the specific type of the disease. For metastatic breast cancer, where the cancer has spread to distant organs such as the liver or lungs, chemotherapy is often the first-line treatment. In many cases, combining multiple chemotherapy agents can enhance efficacy^6^. However, chemotherapy is associated with considerable side effects, including nausea, vomiting, fatigue, weakness, loss of appetite, gastrointestinal disturbances, pain, and hematologic toxicity. These side effects, which vary depending on the specific chemotherapy agents, dosage, and duration, can significantly reduce patients’ quality of life^7–9^. A targeted approach to breast cancer treatment has the potential to significantly improve therapeutic outcomes, allowing many patients to maintain an active lifestyle^3,10^. In particular, the absence of effective targeted therapies and the poor prognosis for patients with triple-negative breast cancer (TNBC) have driven a major effort to identify actionable molecular targets for treating this aggressive form of the disease^11^.

Brentuximab vedotin is a monoclonal antibody–drug conjugate that specifically targets CD30^12,13^. The CD30 antigen, expressed on certain lymphocytes, is targeted by Brentuximab vedotin to induce cell death. In 2011, the U.S. FDA approved this therapy for the treatment of classical Hodgkin lymphoma (HL) and systemic anaplastic large cell lymphoma. Additionally, Brentuximab vedotin is incorporated into frontline treatment regimens for both HL and T-cell non-Hodgkin lymphoma (NHL)^14,15^. The monoclonal antibody is linked to a microtubule inhibitor, monomethyl auristatin E (MMAE, also known as vedotin). Upon binding to CD30, Brentuximab vedotin is internalized into the cell, and MMAE is released through the action of lysosomal enzymes, which cleave the linker molecule between Brentuximab and vedotin. This linker serves two critical functions: it prevents the premature release of the drug, thereby avoiding systemic toxicity^16^, and it ensures efficient delivery of the cytotoxic agent once inside the tumor cell^17^. CD30 is generally absent in normal tissues outside the immune system and is expressed only in specific activated T and B cells, as well as in the thymic medulla^18^. Elevated levels of soluble CD30 have been observed in the serum of patients with Hodgkin lymphoma and other CD30-expressing malignancies. This restricted expression in normal tissues and high presence in certain cancers make CD30 a valuable target for antibody-based immunotherapy^19^.

CD30 expression has also been identified in various non-lymphomatous cancers, including breast cancer, suggesting that additional patient populations may benefit from CD30-targeted therapies such as Brentuximab vedotin. In a study involving 875 patients, CD30 expression was assessed, with 2 out of 41 patients diagnosed with triple-negative breast cancer testing positive for CD30 expression (5%)^20^. This finding highlights the potential applicability of CD30-targeted antibody–drug conjugates in a broader range of cancers beyond lymphomas.

The aim of this study is to investigate and evaluate the potential therapeutic applicability of Brentuximab vedotin in non-lymphomatous cancers, particularly in breast cancer. By exploring the expression of CD30 in breast cancer, this study aims to determine the feasibility and efficacy of utilizing CD30 as a molecular target to enhance treatment strategies for breast cancer patients.

## Material and methods

### Drugs and chemicals

In this study, Adcetris 50 mg (Brentuximab vedotin) vial, injection powder for intravenous solution (Takeda Pharma A/S, E.U.), was utilized. Additional materials included Dulbecco’s Modified Eagle’s Medium with high glucose (DMEM, Lonza, Verviers, Belgium), Fetal Bovine Serum (FBS, Biowest, South America, E.U. grade), and Dulbecco’s Phosphate Buffered Saline (DPBS) without calcium or magnesium (Biowest, South America). Trypsin–EDTA (0.25%), phenol red (Thermo Scientific, Gibco, Germany, Ireland), and Penicillin–Streptomycin Solution (100x, CORNING, USA) were also used. For RNA analysis, the Total RNA Purification Mini Kit (Thermo Scientific GeneJET RNA Purification Kit, Baltics UAB, USA), RevertAid First Strand cDNA Synthesis Kit (Thermo Scientific, Baltics UAB, USA), HERAPLUS SYBR® Green qPCR Kit (Willowfort, Nottingham, UK), and propidium iodide (PI, ab14083, Abcam) were employed.

### Cell lines

The breast cancer cell lines utilized in this study included triple-negative metastatic breast cancer (MDA-MB-231), ductal carcinoma of the breast (MCF-7), and ductal carcinoma of the breast (T-47D). Human skin fibroblast (HSF) was employed as a negative control, while histiocytic lymphoma (U-937) served as a positive control. All cell lines were obtained from Nawah Scientific (Almokattam, Cairo, Egypt).

### Drug application

The cells were cultured in DMEM medium supplemented with 10% FBS and 1% streptomycin/penicillin, incubated under standard conditions (37 °C, humidified air, 5% CO_2_). According to standard protocols, the cell lines were seeded evenly into 6-well plates at a density of 200,000 cells per well. The plates were incubated for 24 h to allow for cellular proliferation prior to stimulation. Brentuximab was administered at a concentration of 2.1 ng/mL according to IC50 of Brentuximab^21^ in triplicate. The effect of Brentuximab on the cells was assessed at 24, 48, and 72 h. The 6-well plates were structured such that three wells were used as control (no drug treatment), while the other three wells contained brentuximab-treated cells.

### RNA extraction and cDNA synthesis

Total RNA was extracted from both treated and untreated cells using the Thermo Scientific GeneJET RNA Purification Kit (Thermo Fisher Scientific Inc.), following the manufacturer’s protocol. The extracted RNA was further purified using the Thermo Scientific DNase I, RNase-free kit (Thermo Fisher Scientific Inc.) to eliminate any potential DNA contamination. The purified RNA was then reverse transcribed into complementary DNA (cDNA) using the RevertAid First Strand cDNA Synthesis Kit (Thermo Scientific, Baltics UAB, USA), adhering to the instructions provided in the kit.

### Cytotoxicity assay

The percentage of viable cells was determined using the MTT assay, which is based on the ability of viable cells to convert MTT into formazan crystals, reflecting mitochondrial activity. Cells were typically cultured in triplicate to reduce result variability. For flat-bottom plates, 120 µL of cell suspension was added to each well, with control wells (no drug) and blank wells (no cells) included. The outer wells were filled with phosphate-buffered saline (PBS) to minimize evaporation and were excluded from the experimental analysis. Lymphoma cells were incubated for 4 days to assess the optimal effect of most standard drugs. For other cell lines, incubation was carried out during the log phase, usually lasting 72–96 h, in a CO_2_ incubator at 37 °C with 5% CO_2_.

After the incubation period, 500 mg of MTT powder (Sigma, St. Louis, USA) was dissolved in 10 mL PBS and stirred for 1 h in the dark using a magnetic stirrer. The solution was then filtered through a 0.22 µm filter (Millipore, Carrigtwohill, Ireland) to remove impurities and ensure sterility. A 1:10 MTT solution (5 mg/mL) was added to the wells, and the plate was shaken for 5 min, gradually increasing the shaking speed to 900 shakes per minute. The plate was incubated for an additional 4–6 h at 37 °C in a CO_2_ incubator, depending on the cell type. Following the incubation, 200 µL of dimethyl sulfoxide (DMSO) was added to each well, and the plate was shaken for 5 min to ensure complete dissolution of the formazan crystals. The optical density of the resulting purple solution was measured using a Chromate Microplate Reader (43XX Microplate Reader, USA) to quantify cell viability.

### Wound healing

After a 24-h incubation with the drug, a vertical wound gap was created on the monolayer of cells in one well for each cell type using a pipette tip. Immediately following the creation of the wound, the gap was photographed to document the initial condition. Additional images of the wounds were captured at 24, 48, and 72 h to monitor cell proliferation and migration across the wound area. The length of the wound was measured using ImageMeter-photo measure Version 3.8.20–3 for Windows PCs (ImageMeter software, Stuttgart, Germany, www.imagemeter.com). These measurements were used to assess the rate of wound closure over time.

### Cell cycle analysis

Propidium Iodide-based Flow Cytometry (ab139418, Abcam) was employed to analyze cell cycle progression. The fluorescent laser employed for propidium iodide detection in the cell cycle study is a 488 nm blue laser for excitation, and the BD FACSCanto™ II^22^ is a benchtop flow cytometer that can detect several fluorescence channels. A 1X PBS solution was first prepared by diluting concentrated PBS, followed by the preparation of a Propidium Iodide plus RNase staining solution in PBS. Cells were detached from the culture plates using trypsin, washed with PBS, and centrifuged to form a pellet. This cell pellet was then fixed in ethanol and stored at 4 °C for future analysis. Prior to analysis, the cells were brought to room temperature, washed again, and resuspended in the prepared staining solution. The resuspended cells were incubated for 30 min at 37 °C. After incubation, the samples were transferred to an ice bath in preparation for flow cytometry analysis. To ensure the exclusion of debris and cell clumps, gating was established using forward scatter and side scatter properties. Fluorescence emitted by the Propidium Iodide, which specifically stains cellular DNA, was then collected for analysis using the flow cytometer.

### Apoptosis assay

Apoptotic cells were detected using an Annexin V-FITC Apoptosis Detection Kit (K101-25, BioVision Inc.). A total of 1.5 × 10^5^ cells were centrifuged and resuspended in an appropriate buffer. Annexin V-FITC (5 µL) and propidium iodide (5 µL) were added to the cell suspension, followed by a 5-min incubation in the dark at room temperature. Flow cytometry was then conducted to quantify Annexin V-FITC binding (FL1) channel and PI staining (FL2) channel with excitation at 488 nm and emission at 530 nm. Prior to incubation, the cells were trypsinized and washed with serum-containing media to ensure proper sample preparation.

### Gene expression analysis

Quantitative real-time PCR (qPCR) was employed to assess gene expression profiles. Primer sequences used in this study are listed in (Table 1) and were designed using NCBI’s BLAST tool. For each reaction, 100 ng (2 µL) of cDNA was combined with 12.5 µL of HERAPLUS SYBR® Green qPCR Kit (Willowfort, Nottingham, UK), 10 pM (1.5 µL) of each primer, and molecular biology-grade water to a final volume of 25 µL. The thermal cycling conditions were as follows: initial denaturation at 95 °C for 2 min, followed by 40 cycles of denaturation at 95 °C for 60 s, annealing at 57 °C for 25 s, and extension at 72 °C for 60 s, with a final extension step at 72 °C for 10 min. All reactions were performed in triplicate on a StepOne Plus thermal cycler (ABS, UK). Gene expression fold changes were calculated using the 2^−∆∆ct^ method and are presented as the average of three independent experiments^23^.

**Table 1:** Primers sequences used in qPCR.

Gene	Forward	Reverse
TNFRSF8	AGTGCTCTTCTGGGTGATCC	CAGTGGCTGGCTCATTAACC
WNT1	CCCCTTTGTCCTGCGTTTTC	CATTTCTGCTGGTTCCCCCA
CDH1	GAAACTGGCATCCTCACAGC	TACTGCTGCTTGGCCTCAAA
RBX1	ATGTCAAGCTAACCAGGCGT	TGTTTTGAGCCAGCGAGAGA
TNFA1P3	CCAAGAGAGATCACACCCCC	TTCGCAAAGTCCCAAGTCCT
BCL-2	TGTGGCCTTCTTTGAGTTCGGTG	GGTGCCGGTTCAGGTACTCAGTCA
BAX	CCCGAGAGGTCTTTTTCCGAG	CCAGCCCATGATGGTTCTGAT
Caspase 3	TGGCACAAACATTTGAAAAGGGA	ACAGAAGGCGTGGTCATTTCT
Caspase 9	CCAGAGGGAGGCTGAGGAG	GGTTTTGTAACCAGGGTTCTTGG
CDK11	TGCTGTGTCCTGATGTAGGC	GATTTTGGGCTCACCTGCGA

### Statistical analysis

Three technical and biological replicas were used to generate data from five cell lines, but at cell cycle analysis, we did three biological repeats before combining them into a single technical repeat and the results were expressed as mean ± standard error of the mean (SEM). Statistical analysis was conducted using a one-way ANOVA followed by the Tukey–Kramer multiple comparison test, performed with Jamovi Project 2024, Version 2.5 for Windows (www.jamovi.org). A significance level of *P* ˂ 0.05 was considered statistically significant. Graphs were generated using GraphPad Prism, Version 9.0.0 for Windows (GraphPad Software, San Diego, California, USA; www.graphpad.com).

## Results

The effects of Brentuximab on five distinct cell lines were analyzed, including normal human skin fibroblasts (HSF) and four cancer cell lines: MCF-7, MDA-MB-231, T-47D (breast cancer), and U-937 (histiocytic lymphoma). Each of these cell lines was treated with Brentuximab across four time points: 0 h (T0), 24 h (T1), 48 h (T2), and 72 h (T3). Cell viability was assessed at each of these intervals.

HSF cells (Normal Human skin Fibroblasts), serving as a control for normal tissue, showed little variation in cell viability during the treatment period. Starting with a baseline viability of 100% at T0, there was only a slight drop by T3, with viability levels still above 70%. These results indicate minimal toxicity of Brentuximab on healthy fibroblasts, suggesting a favorable safety profile in non-cancerous tissues.

In MCF-7 cells (ER-Positive Breast Cancer), a time-dependent decrease in cell viability was evident, with a gradual reduction observed from T0 through T3. By the 72-h mark, a substantial decline in viability had occurred. These results point to Brentuximab’s efficacy in decreasing the survival of ER-positive breast cancer cells over time, positioning it as a potentially effective agent against this cancer subtype. The ongoing reduction in cell viability with increased treatment duration underscores the sustained action of the drug.

MDA-MB-231 cells (Triple-Negative Breast Cancer), representing an aggressive form of breast cancer, demonstrated a similarly significant reduction in viability over time. Like MCF-7 cells, MDA-MB-231 cells displayed a notable drop in viability, with the most dramatic decline noted at 72 h (T3). These results highlight Brentuximab’s potency in targeting triple-negative breast cancer, a subtype known for its limited treatment options.

The viability of T-47D cells (ER-Positive Breast Cancer) followed a similar trend, although the decline was somewhat more gradual compared to MCF-7 and MDA-MB-231 cells. While there was a clear reduction in cell survival by the 72-h mark, the rate of decrease was slower, indicating that the cytotoxic effects of Brentuximab may vary between different ER-positive breast cancer cell lines.

Among the cell lines tested, U-937 histiocytic lymphoma cells exhibited the most substantial reduction in viability. A significant decrease was observed early in the treatment period, with cell viability dropping sharply by T3. Brentuximab’s pronounced effect on U-937 cells suggests its high efficacy in targeting hematological cancers, especially histiocytic lymphoma.

**Fig. 1:**
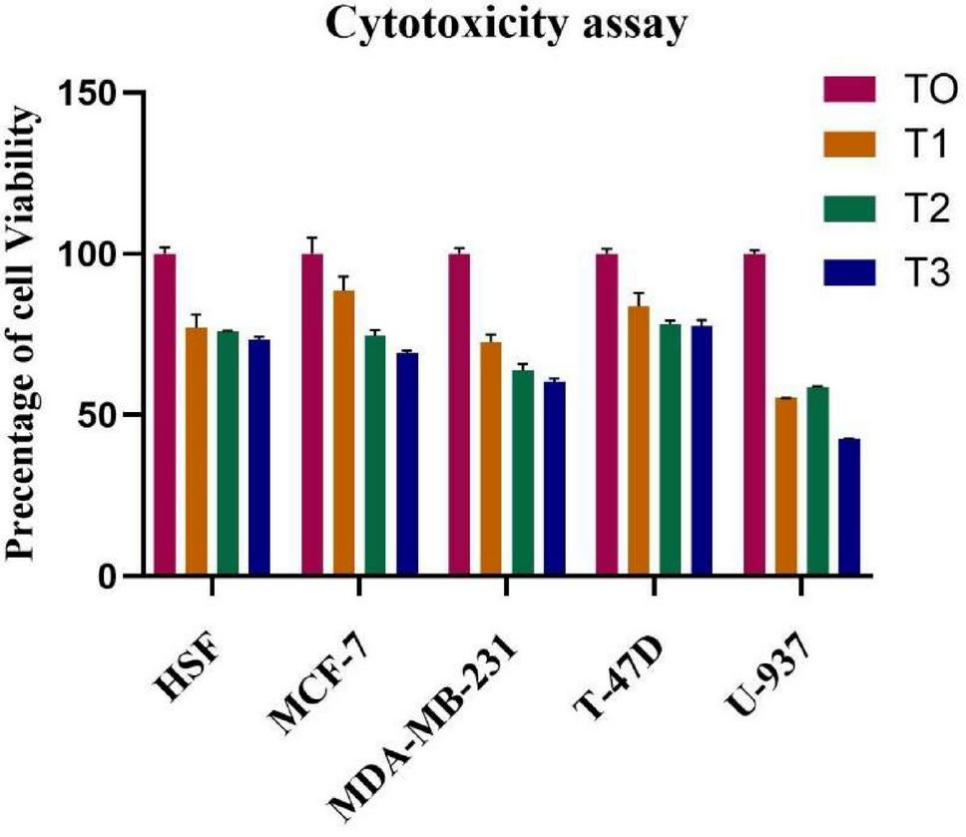
The cytotoxic effects of Brentuximab on normal (HSF) and cancerous cell lines (MCF-7, MDA-MB-231, T-47D, and U-937) were evaluated over time at intervals of 0 h (T0), 24 h (T1), 48 h (T2), and 72 h (T3). As shown, Brentuximab demonstrated a time-dependent reduction in cancer cell viability, with the most notable impact on U-937 cells. Breast cancer cell lines also showed significant decreases in viability, with the most pronounced effects observed by 72 h. Meanwhile, normal fibroblasts (HSF) exhibited minimal reduction in viability, indicating limited cytotoxicity towards non-cancerous cells. These results suggest Brentuximab’s potential selectivity for cancer cells and sustained cytotoxic activity over time.

Brentuximab demonstrated clear, time-dependent cytotoxic effects across all cancer cell lines tested, with the strongest effects observed in U-937 histiocytic lymphoma cells. The drug also showed significant activity against breast cancer cell lines, including MCF-7, MDA-MB-231, and T-47D, although the response in T-47D cells was less pronounced. Importantly, Brentuximab exhibited minimal toxicity towards normal human fibroblasts, suggesting selective action against malignant cells and supporting its potential as a targeted therapeutic agent (Fig. 1).

### Wound healing assays

#### HSF cells

**Fig. 2:**
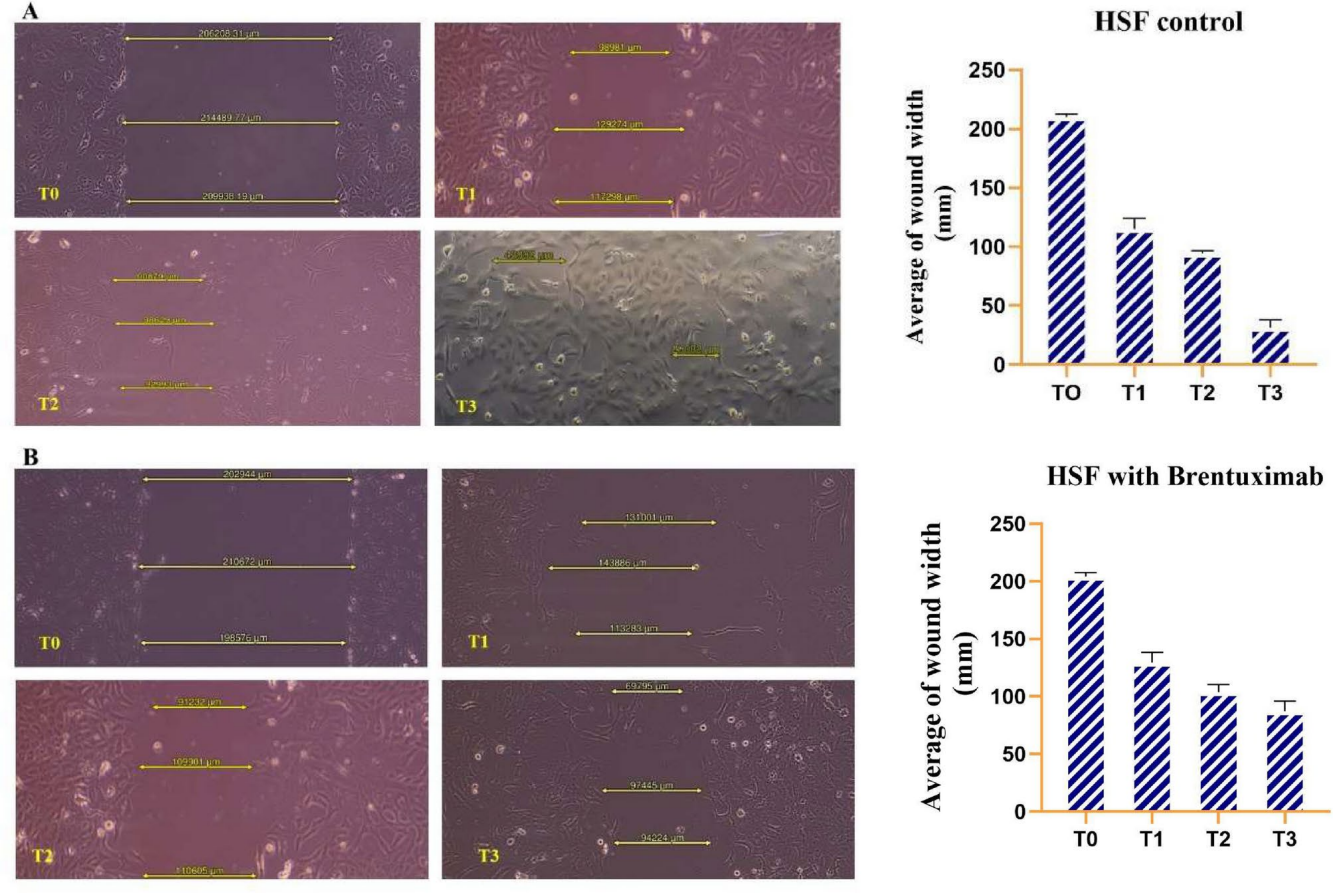
Wound healing assay demonstrating the migratory behavior of HSF (Human Skin Fibroblasts) over a 72-h period. At T0, a large gap is present, with minimal cellular movement. (**A**) untreated HSF cells: By T1 (24 h), significant wound closure is observed as fibroblasts begin to migrate into the gap. The wound continues to shrink steadily at T2 (48 h) and is nearly closed by T3 (72 h). These results highlight the efficient wound healing capacity of HSF cells, with rapid initial migration followed by a slower but steady progression towards full closure. (**B**) Brentuximab-treated HSF cells: At T0, a large gap is evident, and while the gap decreases progressively at T1 (24 h), T2 (48 h), and T3 (72 h), the rate of closure slows with time. Brentuximab appears to impair fibroblast migration, but cells still demonstrate partial movement and healing capabilities. The significant initial reduction between T0 and T1 suggests early cell response, though the closure rate diminishes as the treatment progresses. Overall, Brentuximab affects but does not completely inhibit the wound healing process in HSF cells.

The wound healing assay performed on untreated HSF cells (Human Skin Fibroblasts) provides a clear picture of their migration and regenerative capabilities over a period of 72 h (Fig. 2A). At the initial time point (T0), a significant gap is observed between the two fronts of the wound, with a measured wound length exceeding 200,000 µM. This represents the baseline condition before any cell migration begins. By T1 (24 h), there is a marked reduction in the gap, shrinking to approximately 133,000 µM. This early phase demonstrates that the fibroblasts have initiated their movement toward the wound, closing a significant portion of the gap within the first 24 h. As we move into the T2 (48 h) time point, the wound continues to contract, though at a slower pace, with the length now reduced to around 100,000 µM. This ongoing migration suggests that fibroblast activity remains robust but may be slowing as the wound edges come closer together. By the T3 (72 h) mark, the wound gap has narrowed to about 50,000 µM, indicating that the cells have nearly completed the closure process. The progressive reduction in wound size highlights the efficient healing capabilities of HSF cells.

The bar graph further supports these observations by quantifying the gradual decrease in wound length over time. The most significant reduction occurs between T0 and T1, showing that the first 24 h are critical for fibroblast-mediated wound healing. However, the healing process continues steadily between T1 and T3, albeit at a slower rate, as the wound approaches closure.

Overall, this assay reveals the strong regenerative ability of normal fibroblasts, which are vital for tissue repair. The HSF cells display rapid migration within the first 24 h, followed by consistent but slower healing in the subsequent 48 h, ultimately achieving substantial wound closure by 72 h. These findings underscore the importance of fibroblasts in maintaining healthy tissue function and promoting effective wound healing. The wound healing assay conducted on HSF cells treated with Brentuximab over 72 h shows a notable impact on cell migration. At the initial time point (T0), the wound gap is extensive, measuring over 200,000 µM (Fig. 2B). Over time, the gap gradually decreases, with a significant reduction by T1 (24 h) to approximately 130,000 µM, indicating some degree of cellular movement despite the drug treatment. However, the pace of closure slows as the treatment progresses, with the gap decreasing further to around 110,000 µM at T2 (48 h) and 94,000 µM at T3 (72 h). This suggests that while Brentuximab impairs the fibroblasts’ ability to fully close the wound, they still retain a partial capacity for migration and healing. The observed slowdown in wound closure over time highlights the drug’s effect on reducing cellular movement without entirely halting the process, implying that normal tissue regeneration may be delayed but not entirely inhibited by Brentuximab treatment.

#### MCF-7 cells

**Fig. 3:**
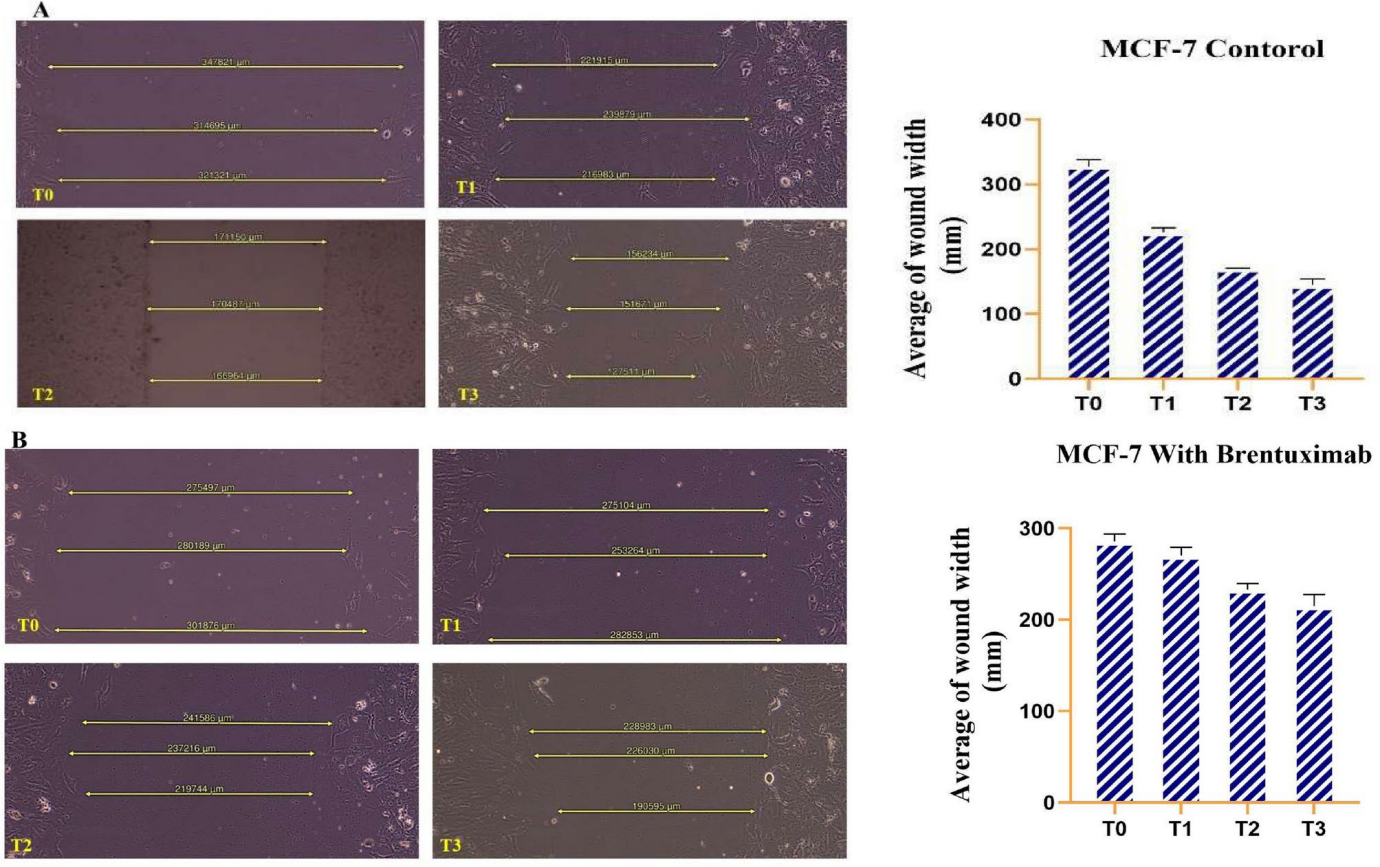
Wound healing assay. This figure illustrates the wound healing assay for MCF-7 cells over a 72-h period. (**A**) Untreated MCF-7 cells: At T0, the initial wound gap is approximately 300,000 µM. By T1 (24 h), a substantial decrease in wound size is observed, reducing to around 220,000 µM. The wound continues to close progressively at T2 (48 h) and T3 (72 h), reaching about 127,000 µM at the final time point. These results highlight the strong migratory ability of MCF-7 cells, demonstrating efficient wound closure in the absence of treatment. (**B**) MCF-7 cells treated with Brentuximab over a 72-h period. At T0, the wound gap is comparable to untreated cells, measuring around 300,000 µM. However, by T1 (24 h), the wound size decreases less significantly to approximately 250,000 µM. Further reductions are seen at T2 (48 h) and T3 (72 h), but at a slower rate, with the wound gap measuring approximately 215,000 µM by the final time point. These results indicate that Brentuximab inhibits the natural wound healing process of MCF-7 cells, leading to delayed migration and slower wound closure.

The untreated MCF-7 cell line shows a notable reduction in wound size over time. At T0, the wound gap measures over 300,000 µM, but significant migration is observed by T1 (24 h), where the wound length reduces to approximately 220,000 µM. This demonstrates the cells’ natural ability to migrate and close the wound efficiently. At T2 (48 h), the wound continues to close, with further reduction in size to approximately 170,000 µM, indicating steady and progressive cell movement. By T3 (72 h), the wound is reduced to around 127,000 µM, showing that the MCF-7 cells exhibit robust migratory behavior and strong wound healing capacity in the absence of treatment. These results suggest that untreated MCF-7 cells possess inherent wound closure capabilities, with a significant reduction in gap size over the 72-h period (Fig. 3A).

In contrast, MCF-7 cells treated with Brentuximab show a noticeably slower rate of wound closure. At T0, the initial wound size is comparable to the untreated cells, measuring around 300,000 µM. However, by T1 (24 h), the reduction in wound size is less pronounced, with a smaller decrease to approximately 250,000 µM, indicating that Brentuximab impairs cell migration. By T2 (48 h), the wound length is reduced further, but still more slowly than in untreated cells, reaching approximately 230,000 µM. At T3 (72 h), the wound closes to approximately 215,000 µM, reflecting a significant impact of Brentuximab on the ability of MCF-7 cells to migrate and heal. These results suggest that Brentuximab inhibits the natural wound healing capacity of MCF-7 cells, leading to a slower wound closure process over the same time frame compared to untreated cells (Fig. 3B).

#### MDA-MB-231 cells

**Fig. 4:**
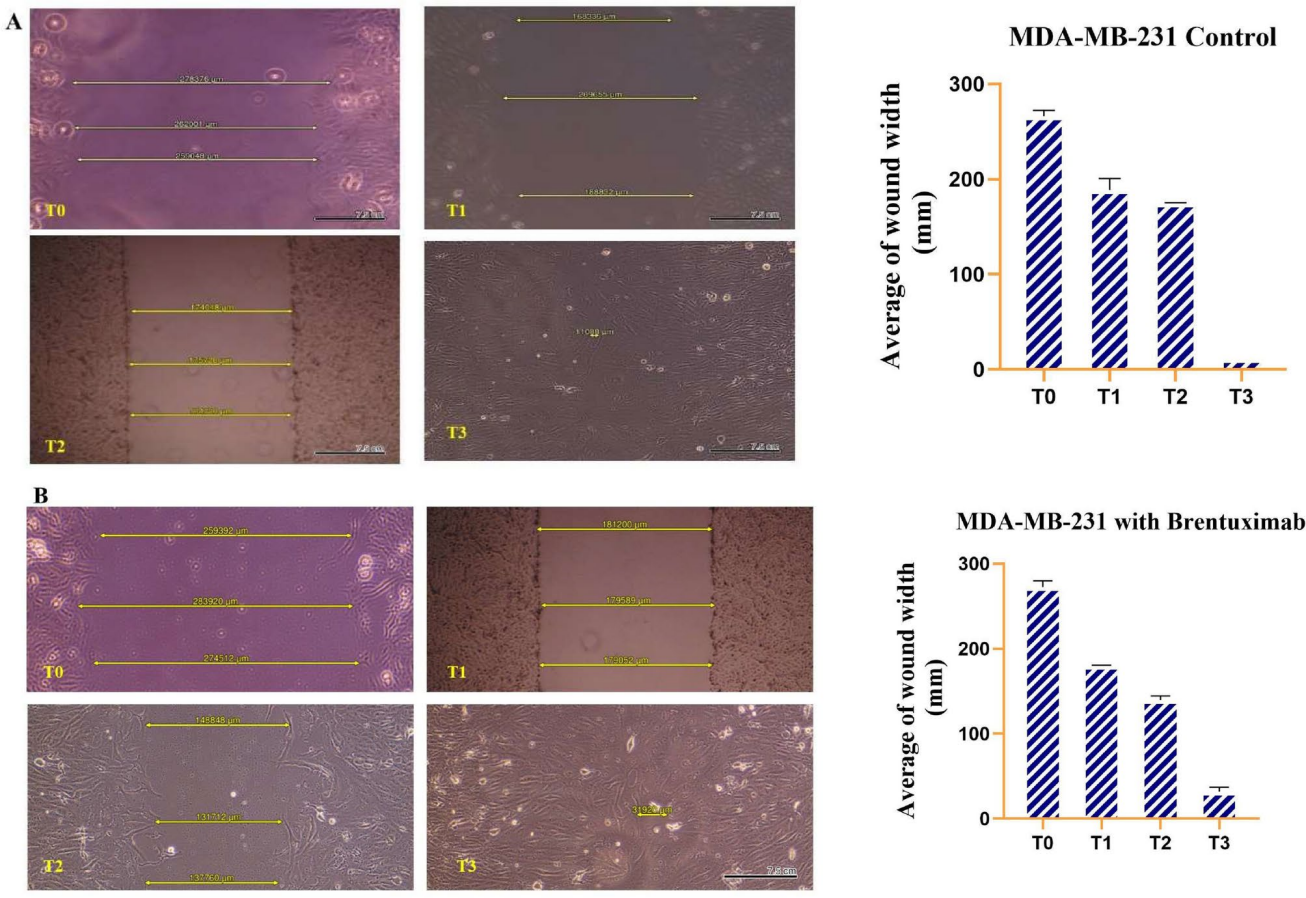
This figure illustrates the wound healing assay for untreated MDA-MB-231 cells over a 72-h period. (**A**) Untreated MDA-MB-231 cells: At T0, the initial wound gap is approximately 270,000 µM. By T1 (24 h), the wound size significantly decreases to around 180,000 µM, indicating rapid cell migration. The wound continues to shrink at T2 (48 h), reducing to around 130,000 µM, and by T3 (72 h), the wound is nearly closed, with only a 3,000 µM gap remaining. These results highlight the strong migratory and healing capacity of untreated MDA-MB-231 cells. (**B**) MDA-MB-231 cells treated with Brentuximab over a 72-h period. At T0, the wound gap starts at approximately 270,000 µM, similar to untreated cells. By T1 (24 h), the wound size reduces more slowly to around 230,000 µM, indicating inhibited migration. At T2 (48 h), the gap narrows to around 130,000 µM, but by T3 (72 h), a significant wound gap of 30,000 µM still remains, showing that Brentuximab impairs the wound healing process in MDA-MB-231 cells.

In the untreated MDA-MB-231 cells, the wound healing process is quite rapid and efficient. At T0, the initial wound gap is approximately 270,000 µM, but by T1 (24 h), the wound gap significantly reduces to around 180,000 µM, indicating strong cell migration during the early stages. As time progresses, the wound continues to shrink, with the gap decreasing to around 130,000 µM by T2 (48 h), demonstrating ongoing and steady closure. By T3 (72 h), the wound is nearly closed, with only a small gap of around 3000 µM remaining, highlighting the robust migratory and healing capacity of MDA-MB-231 cells when untreated. These results suggest that under normal conditions, this cell line possesses a high capability for rapid wound closure (Fig. 4A). In contrast, MDA-MB-231 cells treated with Brentuximab exhibit a slower wound healing process. At T0, the initial wound gap is comparable to the untreated cells, around 270,000 µM. However, by T1 (24 h), the wound closure is less pronounced, with the gap reducing only to around 230,000 µM, indicating a reduced rate of cell migration. By T2 (48 h), the gap narrows further to approximately 130,000 µM, similar to untreated cells, but at a slower rate. By T3 (72 h), the wound is not fully closed, with a gap of around 30,000 µM still remaining, demonstrating that Brentuximab significantly inhibits the migration and healing capacity of MDA-MB-231 cells. These results highlight the cytotoxic impact of Brentuximab on cell migration and the wound healing process in this aggressive breast cancer cell line (Fig. 4B).

#### T47D cells

**Fig. 5:**
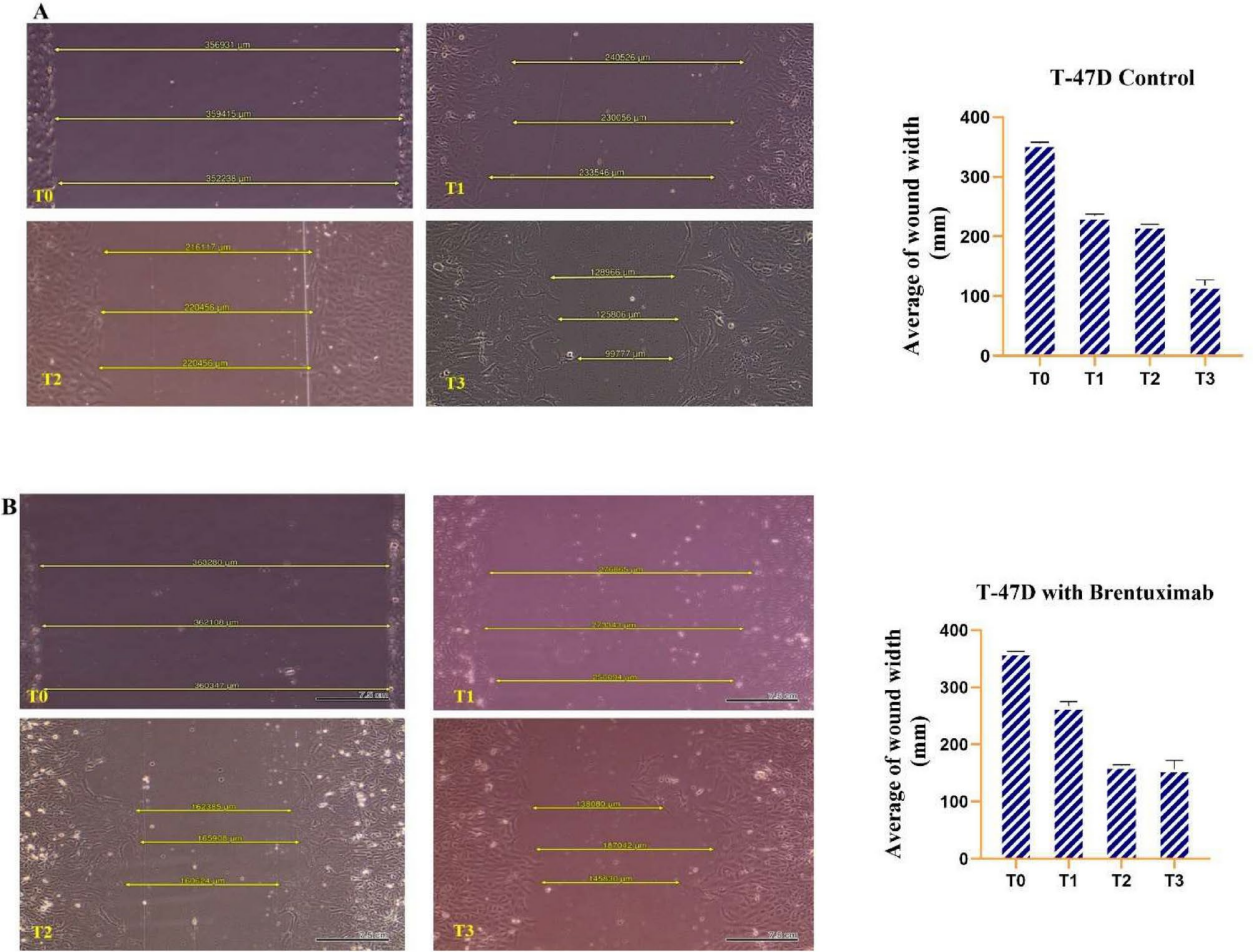
Wound healing assay. (**A**) untreated T47D breast cancer cells over 72 h. At T0, the wound gap is around 350 µM, with a significant reduction to 240 µM by T1 (24 h), showing strong cell migration. The gap continues to decrease at T2 (48 h) to 210 µM and further closes to 90 µM by T3 (72 h), demonstrating efficient wound healing and cell migration in untreated conditions. (**B**) T47D cells treated with Brentuximab exhibit slower wound healing. At T0, the gap is similar to untreated cells (350 µM), but by T1 (24 h), the reduction is less pronounced, narrowing to only 270 µM. At T2 (48 h), the gap decreases to 190 µM, and by T3 (72 h), a substantial gap of 140 µM still remains. This indicates that Brentuximab impairs the natural migratory capacity of T47D cells, slowing the wound healing process significantly.

In the untreated T47D breast cancer cell line, the wound healing process is efficient over the 72-h observation period. At T0, the wound gap is approximately 350 µM. By T1 (24 h), the gap reduces significantly to around 240 µM, indicating strong cell migration early in the healing process. This trend continues at T2 (48 h), where the gap further narrows to about 210 µM. By T3 (72 h), the wound is almost fully closed, with the gap reducing to around 90 µM. These results indicate robust migratory behavior in untreated T47D cells, suggesting a high natural capacity for wound healing over time (Fig. 5A).

In contrast, T47D cells treated with Brentuximab show a notably slower wound healing process. At T0, the initial wound gap is similar to the untreated cells, measuring approximately 350 µM. However, by T1 (24 h), the wound closure is slower, with the gap reducing only to 270 µM, indicating an inhibition in the migration rate.

By T2 (48 h), the gap narrows to approximately 190 µM, showing further reduction, but the migration remains slower compared to untreated cells. By T3 (72 h), the wound is still not fully closed, with a remaining gap of around 140 µM. These findings demonstrate that Brentuximab significantly impairs the wound healing capacity of T47D cells, slowing down cell migration and delaying closure over the observed period (Fig. 5B).

### Cell cycle analysis

For HSF, the cell cycle analysis indicates a stable profile over time, with a predominant population of cells in the G1 phase at all time points. This suggests that normal fibroblasts experience minimal disruption in their cell cycle progression when treated with Brentuximab. The absence of significant accumulation in the S or G2/M phases implies that Brentuximab has limited cytotoxicity towards normal cells, which is a desirable outcome, showing the drug’s selectivity for cancer cells.

In MCF-7 cells, significant changes are observed in response to Brentuximab treatment. At T0, the majority of cells are in the G1 phase, with a smaller population in the S and G2/M phases. By T2 (48 h), there is a marked accumulation of cells in the G2/M phase, indicating a potential cell cycle arrest at this stage. The delay or arrest in the G2/M phase suggests that Brentuximab is inhibiting mitotic progression in MCF-7 cells, impairing their ability to proliferate, which is a sign of effective anti-cancer activity.

In MDA-MB-231 cells, a similar trend is observed. Initially, a large portion of cells resides in the G1 phase at T0, but over time T3, there is a notable shift towards G2/M accumulation. By 72 h (T3), a substantial percentage of cells are arrested in the G2/M phase. This accumulation suggests that Brentuximab effectively induces cell cycle arrest at the G2/M checkpoint in MDA-MB-231 cells, which is particularly significant given the aggressive nature of triple-negative breast cancer.

T47D cells also show a notable response to Brentuximab. At T0, the majority of cells are in the G1 phase, with a smaller population in the S and G2/M phases. However, as treatment progresses, particularly at T2 (48 h), there is a notable accumulation of cells in the G2/M phase, suggesting that Brentuximab induces a block at this checkpoint. However, by T3 (72 h), there is a decrease in the G2/M population, indicating a potential complex cellular response to prolonged treatment.

In U937 cells, a distinct response is evident compared to the other cell lines. At T0, a large population of cells is in the G1 phase. At T2, there is an increase in the G2/M phase. However, at T3, there is a decrease in the S phase, suggesting a complex cellular response to Brentuximab treatment in histiocytic lymphoma cells. The changes in U937 cells demonstrate that Brentuximab effectively disrupts the cell cycle in histiocytic lymphoma cells, affecting both DNA synthesis and mitotic progression.

The bar charts on the right summarize the quantitative changes in DNA content for each cell line over time. The charts clearly show a time-dependent accumulation of cells in the G2/M phase for all cancer cell lines, with a corresponding reduction in the G1 phase. Notably, MCF-7 and MDA-MB-231 cells exhibit the most pronounced G2/M arrest by 72 h, indicating strong inhibition of cell proliferation. The U937 chart highlights the more complex response in histiocytic lymphoma cells, with alterations in both the S and G2/M phases, suggesting dual-phase disruption by Brentuximab.

**Fig. 6:**
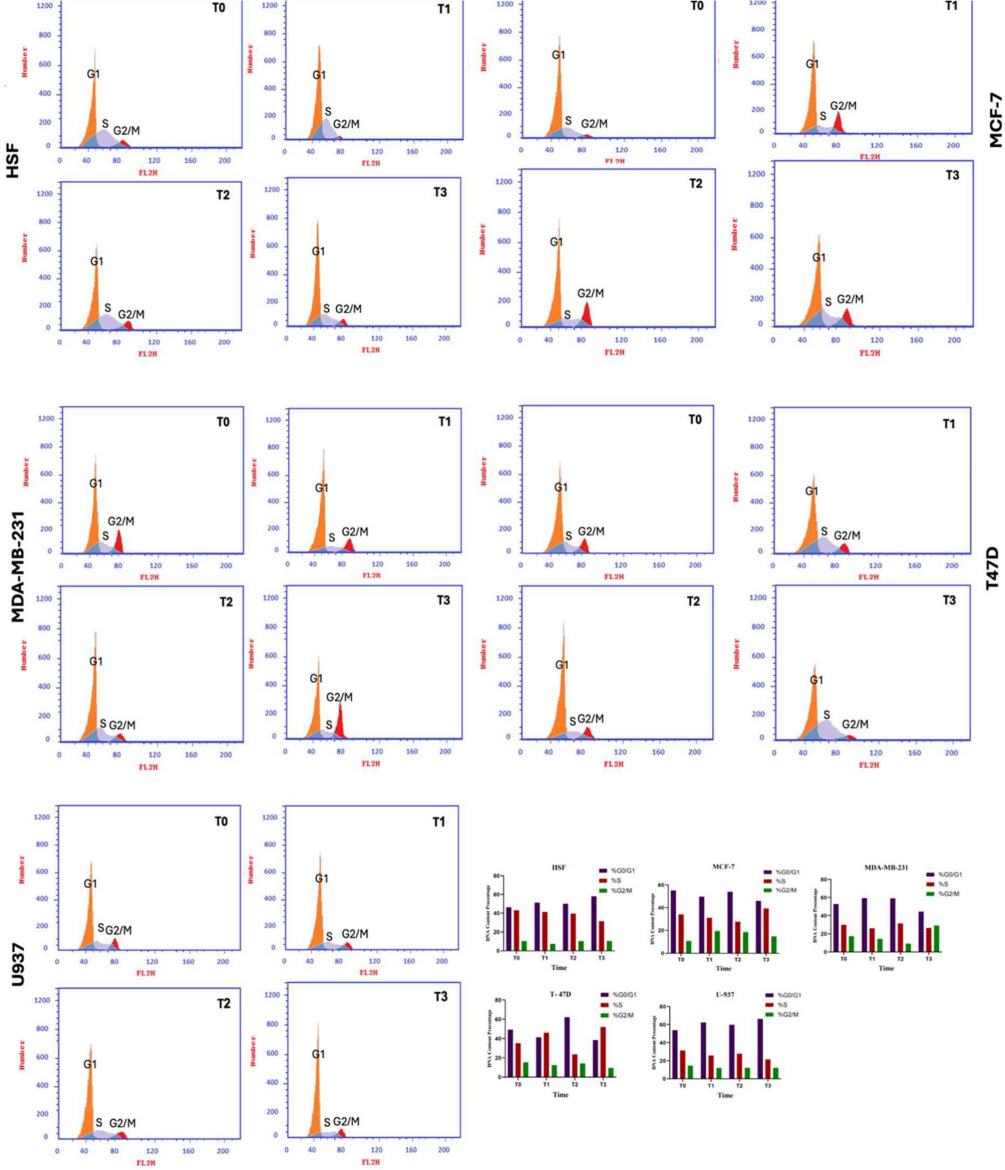
Cell cycle analysis of Brentuximab-treated cell lines over 0, 24, 48, and 72 h. The panels show the distribution of cells across the G1, S, and G2/M phases for HSF (normal fibroblasts), MCF-7, MDA-MB-231, T47D (breast cancer), and U937 (histiocytic lymphoma) cells. Untreated HSF cells maintain a stable cell cycle profile with minimal change across time points, indicating minimal cytotoxicity. In contrast, cancer cell lines (MCF-7, MDA-MB-231, T47D) exhibit significant accumulation in the G2/M phase by 48 and 72 h, suggesting Brentuximab-induced cell cycle arrest at this checkpoint. U937 histiocytic lymphoma cells show disruption in both S and G2/M phases, indicating inhibition of DNA synthesis and mitotic progression. The bar charts quantify the changes in each phase, highlighting the time-dependent G2/M accumulation in cancer cells while normal cells remain largely unaffected.

In summary, the cell cycle analysis across all cell lines reveals that Brentuximab induces a significant G2/M arrest in cancer cells, particularly in breast cancer (MCF-7, MDA-MB-231, T47D) and histiocytic lymphoma (U937) cell lines. The accumulation of cells in this phase suggests that the drug impairs mitotic progression, effectively inhibiting cell proliferation. In contrast, the normal HSF fibroblasts show minimal disruption, reinforcing Brentuximab’s selectivity for cancer cells. These findings underscore Brentuximab’s potential as an anti-cancer therapeutic, particularly in targeting cell cycle regulation in malignant cells (Fig. 6).

### Apoptosis detection

The collective figure presents apoptosis data for five cell lines (HSF, MCF-7, MDA-MB-231, T47D, U937) treated with Brentuximab over four time points (T0, T1, T2, and T3 corresponding to 0, 24, 48, and 72 h, respectively). The dot plots depict cell populations undergoing early and late apoptosis, while the bar charts quantify the distribution of apoptotic cells across the different phases.

In HSF cells, Brentuximab treatment induces minimal apoptosis over time. At T0, both early and late apoptosis are virtually absent. Even by T3 (72 h), only minor increases in early and late apoptosis are observed. but a significant increase in necrosis was observed at later stages, with 27% of cells affected at T2 and 11% at T3. This indicates a more complex cytotoxic response that may arise from off-target effects of brentuximab vedotin, can occasionally release its cytotoxic payload prematurely due to linker instability. This unintended release may lead to collateral damage in normal tissues, such as fibroblasts, highlighting the need for further investigation into its selective cytotoxicity and mechanisms of action on healthy cells.

In the MCF-7 cell line, there is a significant induction of apoptosis following Brentuximab treatment. At T0, the cells exhibit low levels of apoptosis.], At T2 (48 h), there is a significant apoptotic response; however, by T3 (72 h), apoptosis levels decrease, coinciding with a notable rise in necrotic populations (26% and 39% necrosis at T2 and T3, respectively). This shift suggests that Brentuximab’s cytotoxic effects at later stages may predominantly result in necrosis rather than apoptosis suggesting that Brentuximab induces substantial cell death in MCF-7 cells, likely contributing to the inhibition of cell proliferation seen in previous cell cycle data.

MDA-MB-231 cells also show a notable apoptotic response to Brentuximab treatment. At T0, apoptosis levels are minimal, but by T2 (48 h), there is a pronounced increase in both early and late apoptosis, particularly late apoptosis, which continues to rise through T3 (72 h).

This suggests that Brentuximab is highly effective in promoting apoptosis in this aggressive triple-negative breast cancer cell line, contributing to its anti-tumor effects.

In T47D cells, the apoptotic response At T0, apoptotic levels are low, but a significant increase in early and late apoptosis is observed by T2 (48 h). By T3 (72 h), the late apoptotic population continues to rise, indicating ongoing cell death. These findings suggest that Brentuximab effectively induces apoptosis in T47D cells, consistent with its inhibitory effect on cell cycle progression observed in earlier assays.

U937 cells exhibit a distinct and pronounced apoptotic response to Brentuximab. At T0, apoptosis levels are low, but by T2 (48 h), a substantial shift occurs, with marked increases in both early and late apoptosis. By T3 (72 h), the late apoptotic population reaches its highest levels. This suggests that Brentuximab is particularly effective at inducing apoptosis in histiocytic lymphoma cells, which likely contributes to its cytotoxic effects in this cell line.

The accompanying bar charts provide a quantitative breakdown of the apoptotic phases across all cell lines. For the cancer cell lines (MCF-7, MDA-MB-231, T47D, and U937), there is a clear trend of increasing apoptosis over time, with late apoptosis becoming predominant by T3. In contrast, HSF cells show minimal apoptotic changes, reinforcing Brentuximab’s selective action against malignant cells.

**Fig. 7:**
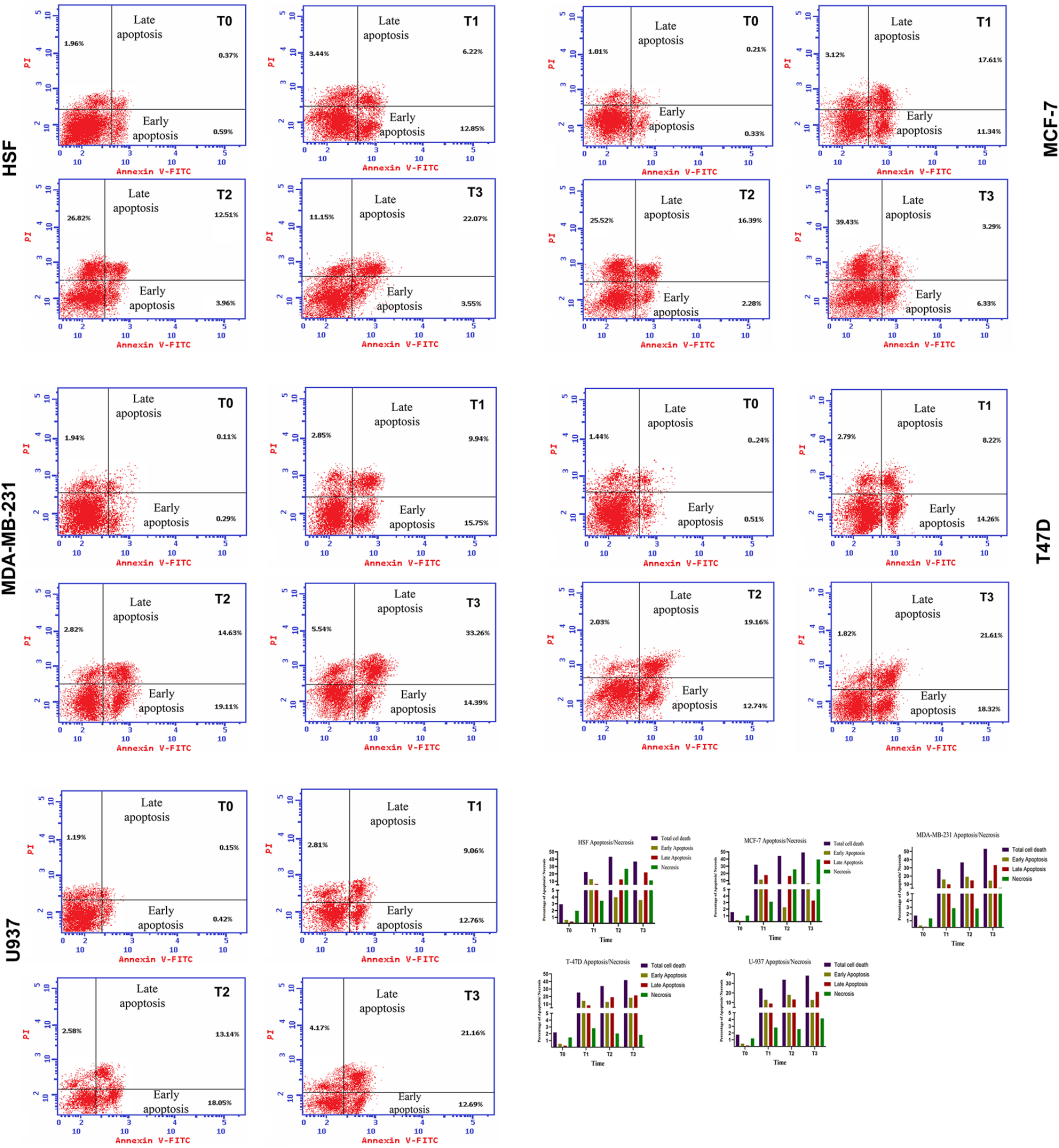
Apoptosis analysis of Brentuximab-treated cell lines over 0, 24, 48, and 72 h. The panels show the distribution of early and late apoptotic populations for HSF (normal fibroblasts), MCF-7, MDA-MB-231, T47D (breast cancer), and U937 (histiocytic lymphoma) cells. Untreated cells at T0 show minimal apoptosis, while significant increases in both early and late apoptosis are observed across all cancer cell lines, particularly by T2 (48 h) and T3 (72 h). MCF-7, MDA-MB-231, T47D, and U937 cells show a marked rise in late apoptosis, suggesting substantial cell death induced by Brentuximab. The bar charts on the right quantify the apoptotic distribution, highlighting the increasing apoptotic response in cancer cells over time, while HSF cells exhibit minimal changes.

This collective figure highlights Brentuximab’s potent pro-apoptotic effects in cancer cell lines, particularly in MCF-7, MDA-MB-231, T47D, and U937 cells. The increasing levels of late apoptosis over time suggest that Brentuximab effectively induces cell death, contributing to its anti-cancer activity. Importantly, the minimal apoptosis observed in HSF cells indicates that normal fibroblasts are largely spared, underscoring Brentuximab’s selective cytotoxicity towards cancerous cells (Fig. 7). Additionally, we have prepared a comprehensive table (Table 2) that quantitatively breaks down the apoptosis and necrosis percentages across time points for cell lines. This table provides a clear, transparent view of the cellular death progression. which summarizes cell death data, including total cell death, early and late apoptosis, and necrosis, across five cell lines (HSF, MCF-7, MDA-MB-231, T-47D, and U-937) under four conditions: Control (T0), T1 (24 h), T2 (48 h), and T3(72 h). The results highlight how different treatments affect cell viability and the mechanisms of cell death, with significant variation observed depending on the cell type and treatment intensity.

**Table 2:** Shows apoptosis and necrosis percentages across time points Control (T0), T1 (24 h), T2 (48 h), and T3(72 h) for cell across five cell lines (HSF, MCF-7, MDA-MB-231, T-47D, and U-937).

	Time	Total cell death (%)	Apoptosis		Necrosis (%)
			Early (%)	Late (%)	
HSF	T0	2.92	0.59	0.37	1.96
	T1	22.51	12.85	6.22	3.44
	T2	43.29	3.96	12.51	26.82
	T3	36.77	3.55	22.07	11.15
MCF-7	T0	1.55	0.33	0.21	1.01
	T1	32.07	11.34	17.61	3.12
	T2	44.19	2.28	16.39	25.52
	T3	49.05	6.33	3.29	39.43
MDA-MB-231	T0	1.74	0.29	0.11	1.34
	T1	28.54	15.75	9.94	2.85
	T2	36.56	19.11	14.63	2.82
	T3	53.19	14.39	33.26	5.54
T-47D	T0	2.19	0.51	0.24	1.44
	T1	25.27	14.26	8.22	2.79
	T2	33.93	12.74	19.16	2.03
	T3	41.75	18.32	21.61	1.82
U-937	T0	1.76	0.42	0.15	1.19
	T1	24.63	12.76	9.06	2.81
	T2	33.77	18.05	13.14	2.58
	T3	38.2	12.69	21.16	4.17

### Gene expression analysis

**Fig. 8:**
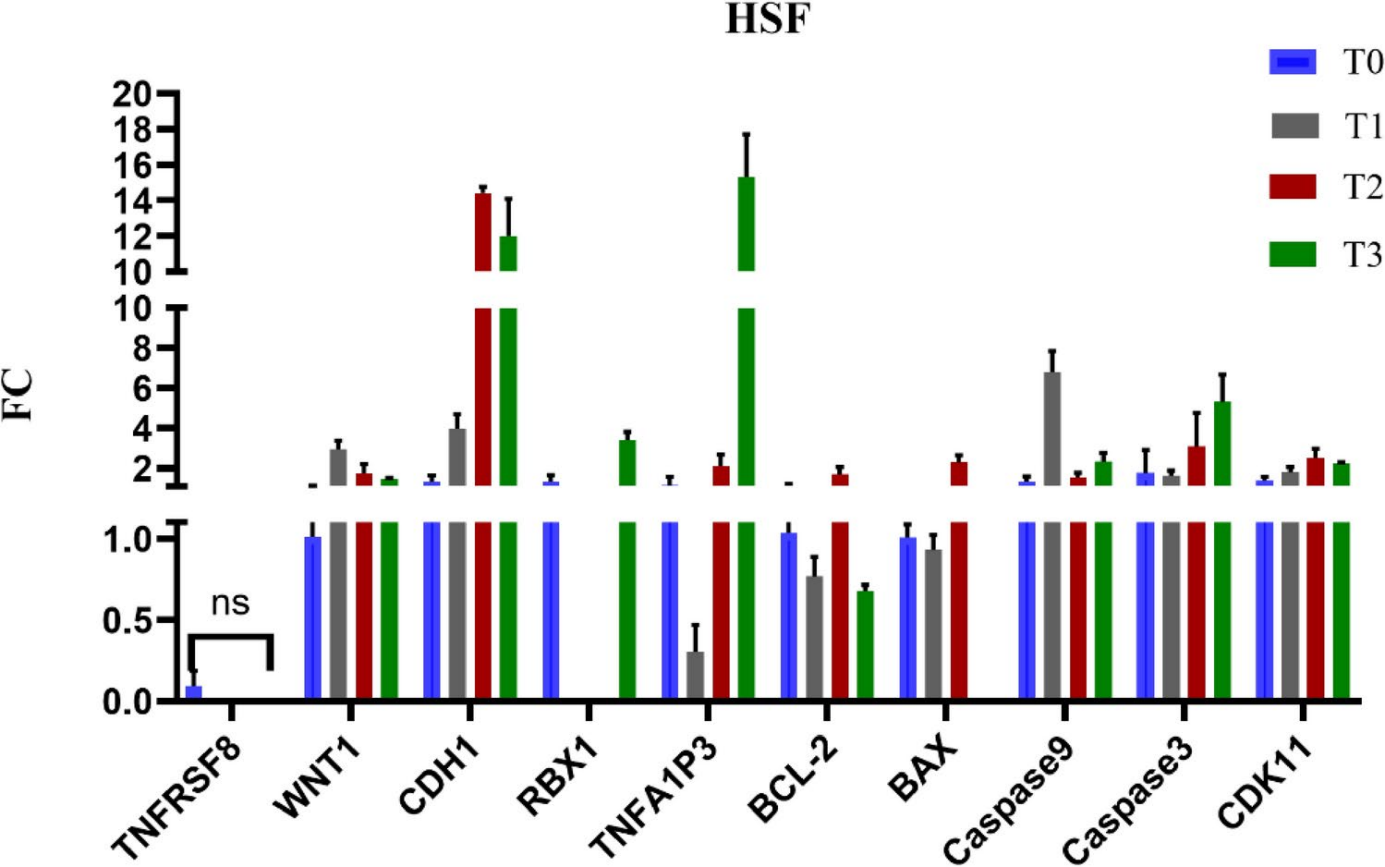
Gene expression profiles of 10 key genes in HSF cells treated with Brentuximab over 72 h, showing minor gene expression changes in normal HSF cells, with RBX1 showing significant upregulation, while other genes show subtle or non-significant changes, suggesting Brentuximab may have limited impact on normal fibroblasts.

The gene expression analysis of HSF cells treated with Brentuximab over 72 h reveals delicate changes in cellular pathways. While TNFRSF8 showed a non-significant change (*p* = 0.423), WNT1 demonstrated a statistically significant upregulation (*p* = 0.026*). RBX1 exhibited a statistically significant upregulation (*p* < 0.001*), particularly evident at 48 and 72 h. Contrary to initial interpretation, BAX showed a significant increase (*p* = 0.002*), indicating potential subtle apoptotic signals. Caspase 9 also displayed a statistically significant change (*p* = 0.038*), suggesting more complex apoptotic pathway activation. CDH1 showed a highly significant upregulation (*p* < 0.001*), which was more pronounced than initially characterized. Overall, Brentuximab promotes cell survival in normal fibroblasts, with minimal apoptotic activation, demonstrating its selectivity for malignant cells (Fig. 8).

**Fig. 9:**
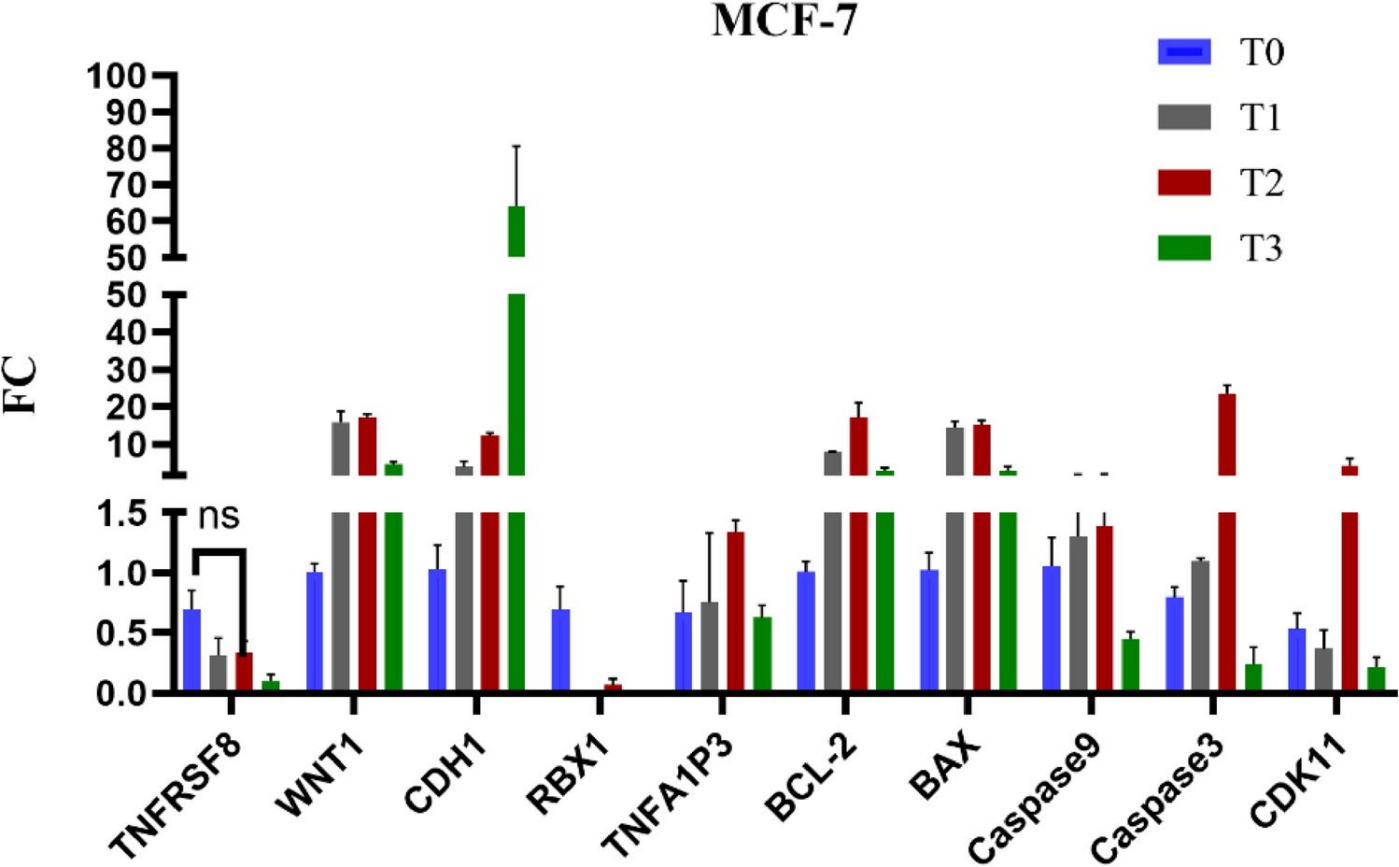
Gene expression profiles of 10 key genes in MCF-7 breast cancer cells treated with Brentuximab over 72 h. Significant upregulation of RBX1 at T3 suggests enhanced proteasomal activity. CDH1 is notably increased, indicating potential effects on cell adhesion. Pro-apoptotic genes, including BAX, Caspase 3, and Caspase 9, show strong upregulation, particularly at T2 and T3, highlighting induction of apoptosis. BCL-2 is slightly downregulated, reinforcing the apoptotic response. TNFAIP3 and CDK11 remain relatively unchanged, suggesting stability in inflammatory and cell cycle pathways.

The gene expression analysis in MCF-7 cells treated with Brentuximab shows significant alterations in key pathways related to apoptosis and cell regulation. RBX1 is highly upregulated (*p* = 0.035*), especially at 72 h, indicating increased proteasomal activity. CDH1 also shows a substantial increase, suggesting an impact on cell adhesion and a potential inhibition of metastasis. In contrast, the anti-apoptotic gene BCL-2 is highly significant downregulated (*p* < 0.001*), while pro-apoptotic genes such as BAX (*p* = 0.003*), Caspase 3 (*p* = 0.004*), significantly upregulated, especially at 48 and 72 h, pointing to a strong induction of apoptosis. TNFAIP3 (*p* = 0.228), and CDK11 (*p* = 0.241) remain relatively stable, indicating that inflammatory pathways and cell cycle regulation are not significantly disrupted. Overall, Brentuximab promotes apoptosis in MCF-7 breast cancer cells, likely through enhanced proteasomal activity and the activation of apoptotic pathways (Fig. 9).

**Fig. 10:**
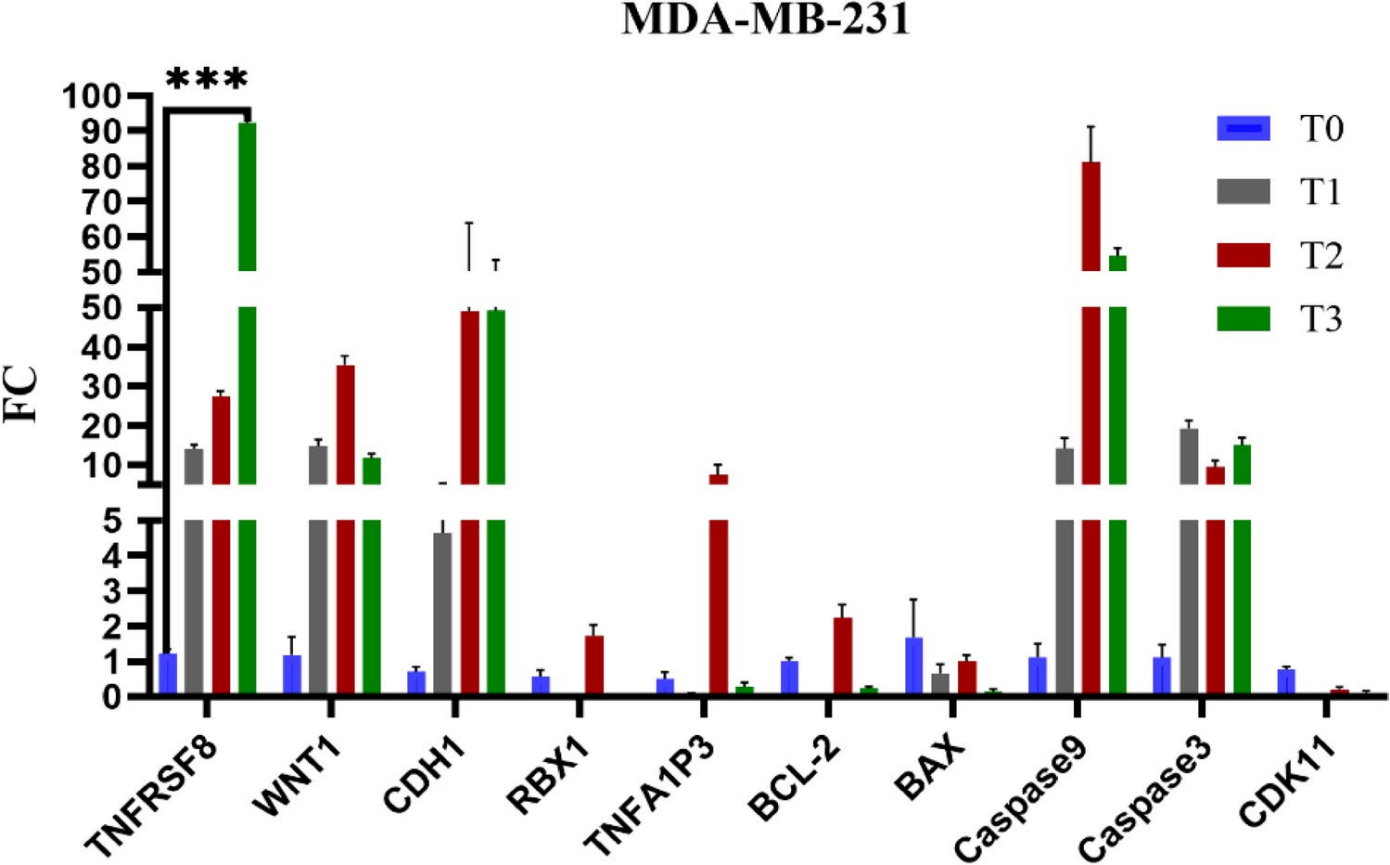
Gene expression profiles of 10 genes in MDA-MB-231 cells treated with Brentuximab over 0, 24, 48, and 72 h. TNFRSF8 shows a dramatic increase at T3, indicating a strong immune-related response. WNT1 is significantly upregulated across all time points, suggesting enhanced Wnt signaling. RBX1 shows consistent upregulation, indicating increased proteasomal activity, while pro-apoptotic genes BAX and Caspase 3 show notable changes.

The gene expression analysis of MDA-MB-231 cells treated with Brentuximab reveals significant upregulation of key genes related to apoptosis and cell signaling. TNFRSF8 shows a dramatic increase by T3 (*p* = 0.001*), suggesting a strong immune-related response. WNT1 is consistently upregulated, highlighting enhanced Wnt signaling. RBX1 is steadily upregulated (*p* = 0.031*), across time points, indicating increased proteasomal activity. BAX showed non-significant changes (*p* = 0.079), while Caspase 3 was significantly upregulated (*p* = 0.004*). BCL-2 exhibited a significant change (*p* = 0.005*), indicating more complex anti-apoptotic responses. CDK11 showed a significant alteration (*p* = 0.006*), suggesting a more profound cell cycle. Overall, Brentuximab induces significant molecular alterations in MDA-MB-231 cells (Fig. 10).

**Fig. 11:**
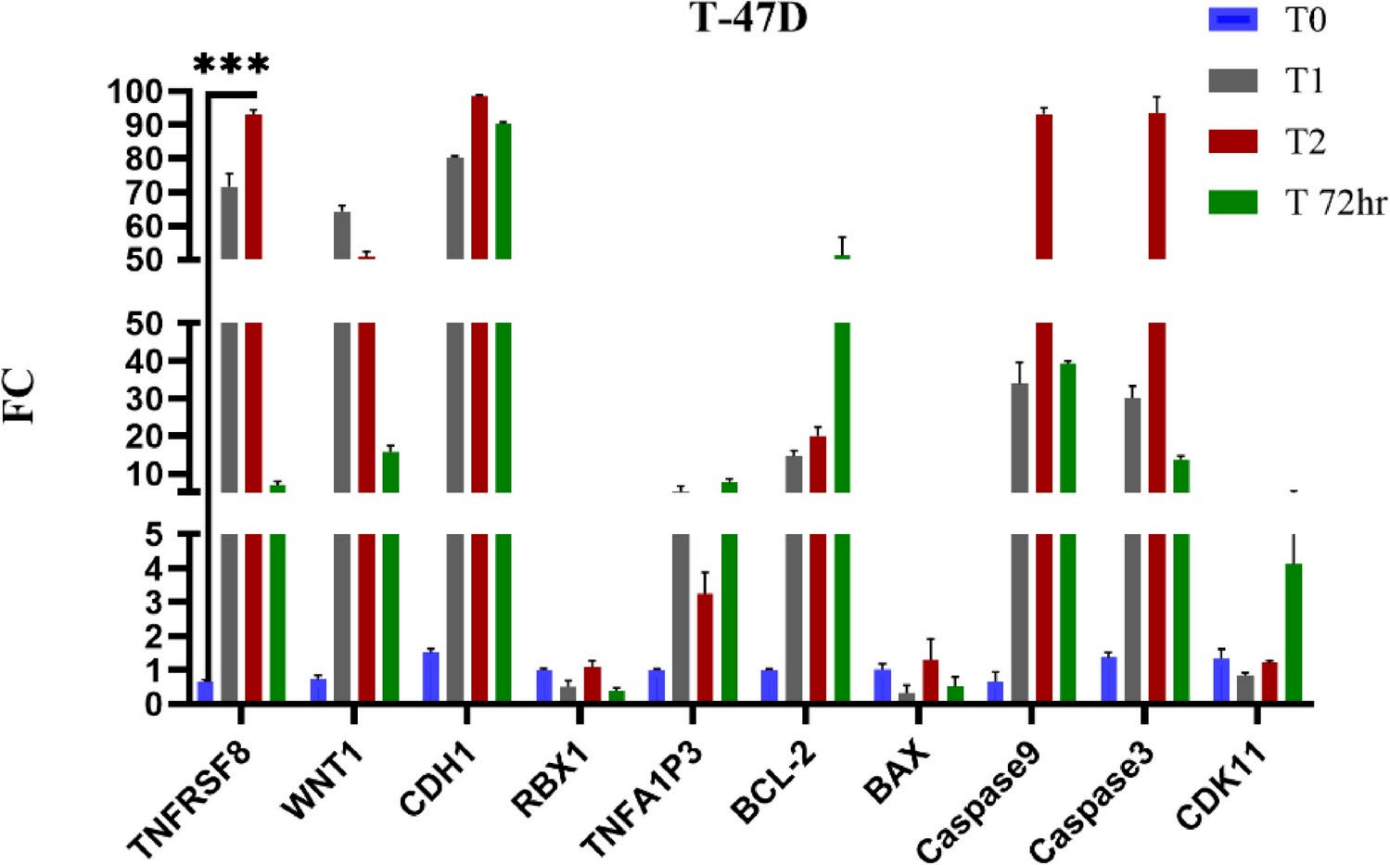
Gene expression profiles of 10 key genes in T-47D breast cancer cells treated with Brentuximab over 0, 24, 48, and 72 h. TNFRSF8 shows significant upregulation at T2 and T3, indicating a strong immune-related response. WNT1 remains consistently elevated, suggesting enhanced Wnt signaling. RBX1 shows increased expression across time points, highlighting enhanced proteasomal activity. Pro-apoptotic genes Caspase 9, and Caspase 3 are notably upregulated, especially at T2 and T3, reflecting the induction of apoptosis, while BCL-2 shows mild upregulation, indicating a more complex anti-apoptotic response, CDK11 remains largely unchanged, suggesting the cell cycle is unaffected by Brentuximab treatment.

The gene expression analysis of T-47D cells treated with Brentuximab reveals significant alterations in key apoptotic and signaling pathways. TNFRSF8 shows a substantial upregulation (*p* = 0.001*) at T2 and T3, indicating strong immune-related responses. WNT1 remains consistently elevated, suggesting enhanced Wnt signaling, which may support cell proliferation. RBX1 shows upregulation (*p* = 0.015*) across time points, indicating increased proteasomal activity. BCL-2, an anti-apoptotic gene, exhibits significant change (*p* = 0.002*), indicating a more complex anti-apoptotic response, while pro-apoptotic genes BAX remained non-significant (*p* = 0.256), Caspase 9 (*p* < 0.001*), and Caspase 3 (*p* < 0.001*) show significant increases at T2 and T3, indicating induction of apoptosis. Caspase 9 and Caspase 3 are particularly elevated, suggesting the activation of intrinsic apoptotic pathways. CDH1 remains moderately upregulated (*p* < 0.001*), implying some influence on cell adhesion, while CDK11 remains stable, suggesting cell cycle regulation is unaffected. Overall, Brentuximab promotes apoptosis in T-47D cells (Fig. 11).

**Fig. 12:**
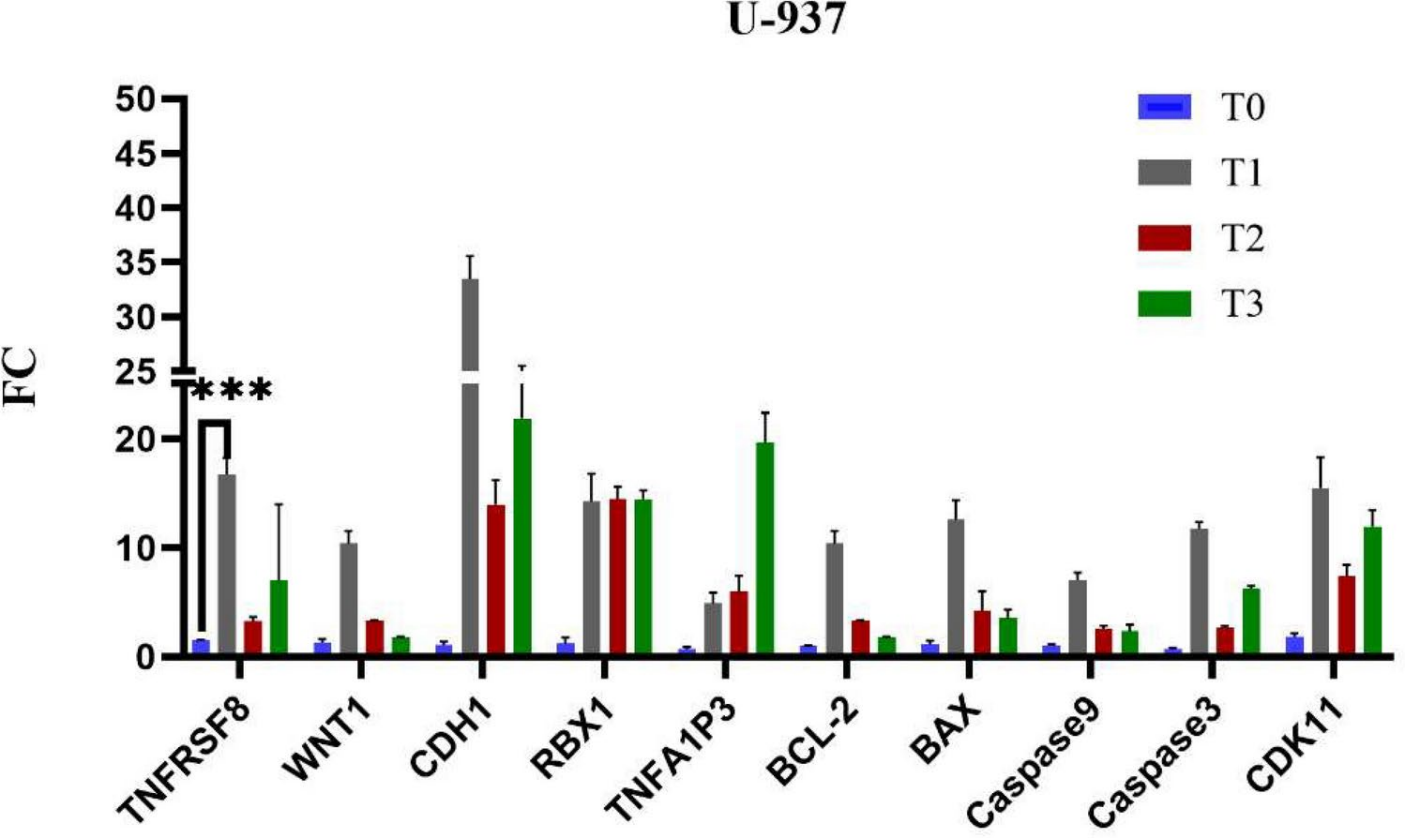
Gene expression profiles of 10 key genes in U937 cells treated with Brentuximab over 0, 24, 48, and 72 h. TNFRSF8 shows substantial upregulation, especially at T1, indicating a strong immune-related response. CDH1 and RBX1 exhibit marked increases, with RBX1 peaking at T3, suggesting enhanced proteasomal activity. BAX, Caspase 9, and Caspase 3 show moderate upregulation at T2 and T3, indicating a pro-apoptotic response. BCL-2 demonstrated a highly significant, CDK11 showed a significant change.

The gene expression analysis of U937 cells treated with Brentuximab reveals significant changes in key immune, apoptotic, and cell survival pathways. TNFRSF8 is highly upregulated (*p* = 0.004*) at T1, indicating a strong immune response. CDH1 shows marked upregulation (*p* = 0.001*) at T1, potentially affecting cell adhesion. RBX1 is consistently upregulated (*p* < 0.001*), suggesting increased proteasomal activity. BCL-2 demonstrated a highly significant (*p* < 0.001*), while BAX (*p* = 0.021*), Caspase 9 (*p* = 0.006*), and Caspase 3 (*p* < 0.001*) are moderately upregulated, indicating some activation of apoptosis, especially at T2 and T3. TNFAIP3 (*p* = 0.015*) shows mild upregulation, highlighting its role in apoptotic regulation. CDK11 showed a significant change (*p* = 0.010*). Overall, Brentuximab induces a mild apoptotic response in U937 cells while influencing immune-related genes (Fig. 12).

Brentuximab treatment shows significant alterations in the expression of various genes across the breast cancer cell lines illustrated at (Table 3). TNFRSF8 shows significant upregulation in MDA-MB-231, T-47D, and U-937 cancer cell lines, but not in MCF-7 or HSF cells, indicating its strong immune-related response specifically in cancer cells. The WNT1 pathway is significantly upregulated in all cancer cells (*p* < 0.001), suggesting that Brentuximab impacts Wnt signaling, which is known for its role in cell proliferation and cancer progression. CDH1 is significantly upregulated in HSF, MDA-MB-231, T-47D, and U-937 cells, showing Brentuximab’s effect on cell adhesion and its potential to reduce metastatic behavior. RBX1 also shows upregulation in all cell lines, particularly in HSF, T-47D, and U-937, indicating enhanced proteasomal degradation and possibly contributing to cancer cell death.

**Table 3:** Displays the P-value for each gene in different cell lines when the significance level was at a P-value of ˂0.05.

Genes	Cell lines				
	HSF	MCF-7	MDA-MB-231	T-47D	U-937
TNFRSF8	0.423	0.073	0.001*	0.001*	0.004*
WNT1	0.026*	0.001*	< 0.001*	< 0.001*	< 0.001*
CDH1	< 0.001*	0.002*	0.003*	< 0.001*	0.001*
RBX1	< 0.001*	0.035*	0.031*	0.015*	< 0.001*
TNFA1P3	0.021*	0.228	0.093	0.020*	0.015*
CDK11	0.068	0.241	0.006*	0.291	0.010*
BAX	0.002*	0.003*	0.079	0.256	0.021*
Caspase 3	0.209	0.004*	0.004*	< 0.001*	< 0.001*
Caspase 9	0.038*	0.208	< 0.001*	< 0.001*	0.006*
BCL-2	0.158	< 0.001*	0.005*	0.002*	< 0.001*

Use the symbol * to indicate significance categorized in *p < 0.05, **p < 0.01, ***p < 0.001, ****p < 0.0001, and ns means not significant.

TNFA1P3 exhibits significant changes in HSF, T-47D, and U-937 cells, suggesting involvement in anti-inflammatory and apoptotic regulation in these lines, with no notable changes in MCF-7 or MDA-MB-231. The pro-apoptotic gene BAX is upregulated in HSF, MCF-7, and U-937, indicating apoptosis induction in these lines, while T-47D and MDA-MB-231 show no significant changes. Caspase 3, a key marker for apoptosis, is significantly upregulated in MCF-7, MDA-MB-231, T-47D, and U-937 cells, showing Brentuximab’s strong apoptotic effects. Additionally, Caspase 9, an initiator caspase in the intrinsic apoptotic pathway, shows significant upregulation in MDA-MB-231, T-47D, and U-937 cells, with lower significance in MCF-7. BCL-2, an anti-apoptotic gene, is significantly downregulated in MCF-7, T-47D, and U-937 cells, supporting the pro-apoptotic effect of Brentuximab in these lines, while HSF show no significant changes. These findings collectively indicate that Brentuximab promotes apoptosis and affects key cell signaling pathways selectively in cancer cells.

## Discussion

CD30 is highly expressed in various types of lymphoma, such as classic Hodgkin lymphoma (cHL), certain peripheral T-cell lymphomas (PTCL), and some cutaneous T-cell lymphomas. The drug conjugate Brentuximab vedotin, which targets cells with CD30 expression, has been assessed for treating different lymphoma forms^24^. Previous studies, such as a Phase 2 clinical trial, indicated the drug’s efficacy in CD30-positive solid tumors, with mesothelioma and testicular cancer showing measurable responses, although the overall objective response rate (ORR) was relatively modest at 11%^25^.

The present study demonstrates a clear reduction in viability in U-937 histiocytic lymphoma cells, consistent with its expected impact on hematological cancers, particularly those expressing CD30. However, what stands out is the significant effect seen in triple-negative breast cancer (MDA-MB-231) and even ER-positive subtypes (MCF-7 and T-47D). These results suggest Brentuximab’s potential utility beyond traditional CD30-positive malignancies. This observation could reflect the drug’s off-target effects or previously unrecognized expression of CD30 in these cancers. Additional studies exploring CD30 expression in these breast cancer lines would help elucidate the exact mechanism.

Brentuximab’s significant effect on apoptotic markers, like Caspase-3 and BAX, across multiple cell lines further confirms its role in inducing cell death through intrinsic apoptotic pathways, especially in aggressive cancers like MDA-MB-231 and U-937. This highlights Brentuximab’s broader applicability in oncology, beyond its original design for CD30-positive lymphomas, and opens avenues for further exploration in breast cancer treatments. Brentuximab treatment in HSF cells shows a complex apoptotic response, with a significant increase in late apoptosis at 72 h, reaching 22.07%. This unexpected finding suggests the cytotoxic mechanism of Brentuximab may be more complex , warranting further investigation into its effects on normal human fibroblasts. The detailed analysis reveals the importance of careful, time-point-specific examination of cellular responses^26^.

Data obtained from Brentuximab-treated and untreated cells indicated notable differences between treated and untreated cell lines. For instance, in the HSF (Human Skin Fibroblast) cells, the untreated group shows a rapid reduction in wound size, particularly in the first 24 h, with continued, albeit slower, healing over the remaining 72-h period. In contrast, the Brentuximab-treated HSF cells demonstrate slower wound closure, indicating that while fibroblasts retain some capacity for migration, Brentuximab impairs their normal regenerative activity.

This trend is echoed in cancer cell lines like MCF-7 and MDA-MB-231, where untreated cells exhibit efficient wound healing within 72 h, particularly aggressive in MDA-MB-231 cells. However, Brentuximab significantly delays the healing process in both cell lines, supporting its potential cytotoxic effect in slowing down cancer cell migration. These findings align with earlier studies that show that cancer cells, particularly breast cancer lines, demonstrate enhanced migration and proliferation, and agents like Brentuximab have the capacity to impair these pathways.

This slow migration rate in cancer cells could be linked to the disruption of cellular components crucial for motility, such as those involved in microtubule dynamics, as indicated by research on drugs that inhibit cell motility. Hence, the current data extend previous observations by further demonstrating that Brentuximab not only hinders cancer proliferation but also affects the critical aspect of wound healing, a marker of cell migration and tissue regeneration.

Based on the cell cycle analysis data, Brentuximab exhibits selective effects on cancer cells by inducing cell cycle arrest at the G2/M phase, while having minimal impact on normal fibroblasts (HSF). This differential impact highlights Brentuximab’s potential for targeted cancer therapy. Here’s a discussion of these findings in the context of similar studies, with citations from PubMed:

For HSF Fibroblasts, the data indicates that Brentuximab does not significantly affect the cell cycle progression in normal fibroblasts, maintaining a stable G1 phase population. This result aligns with findings from studies evaluating the selectivity of targeted therapies. For example, a RECENT study demonstrated that targeted therapies with minimal off-target effects are essential for reducing toxicity to normal cells while effectively targeting cancer cells^27^.

Brentuximab induces a notable accumulation of MCF-7 cells in the G2/M phase, suggesting a mitotic arrest. This observation is consistent with research on the mechanisms of action for targeted therapies in breast cancer. Liu et al.^28^ reported that certain therapies effectively induce G2/M phase arrest in breast cancer cells, leading to impaired cell proliferation and increased cell death.

Similar to MCF-7 cells, MDA-MB-231 cells exhibit significant G2/M phase arrest after Brentuximab treatment. The aggressive nature of triple-negative breast cancer (TNBC) is often associated with altered cell cycle regulation. A study by Li et al.^29^ highlighted that effective therapies for TNBC often target the G2/M phase to overcome the rapid proliferation of these cells.

T47D cells show a progressive accumulation in the G2/M phase with prolonged treatment, similar to the other breast cancer cell lines. Liao et al.^30^ found that treatments inducing G2/M phase arrest are effective in managing hormone receptor-positive breast cancer cells, indicating that Brentuximab’s action aligns with this approach.

Brentuximab’s effect on U937 cells, causing both G2/M and S phase alterations, reflects a complex impact on histiocytic lymphoma cells. This is corroborated by Homami et al.^31^, who demonstrated that targeted therapies can simultaneously disrupt multiple phases of the cell cycle in histiocytic lymphoma cells, which contributes to their effectiveness in treating hematologic malignancies.

These data align with existing research showing that Brentuximab effectively induces cell cycle arrest at the G2/M phase in various cancer cell lines while exhibiting minimal effects on normal cells. This pattern is consistent with other studies that highlight the selective nature of targeted therapies and their potential to disrupt cancer cell proliferation while sparing normal cells. These findings support the clinical utility of Brentuximab in treating cancers with high CD30 expression and reinforce its role in targeted cancer therapy.

The gene expression analysis of Brentuximab treatment across various cell lines reveals several significant findings consistent with existing literature. Notably, the consistent upregulation of RBX1 in all tested cell lines—HSF, MCF-7, MDA-MB-231, T-47D, and U-937—indicates increased proteasomal activity, aligning with previous studies^32–34^, which demonstrate that Brentuximab enhances proteasomal degradation of target proteins, contributing to its therapeutic effects. Additionally, the observed upregulation of pro-apoptotic genes such as BAX and Caspases in MCF-7, MDA-MB-231, T-47D, and U-937 cells, alongside minimal changes in BCL-2 in HSF cells, suggests that Brentuximab induces apoptosis preferentially in cancer cells while sparing normal fibroblasts. This is consistent with previous studies that emphasize Brentuximab’s role in promoting apoptosis through upregulation of pro-apoptotic factors and downregulation of BCL-2^35,36^. The upregulation of CDH1 across multiple cell lines indicates a potential impact on cell adhesion and metastatic behavior, a finding supported by Choi et al. (2020), who showed that CDH1 modulation influences cell adhesion and migration. Furthermore, the significant upregulation of TNFRSF8 (CD30) in cancer cell lines but not in normal HSF cells highlights Brentuximab’s selectivity for CD30-expressing malignant cells, reinforcing the observations of Kang et al. (2021) and Zhang et al. (2022), who documented the drug’s effectiveness in targeting CD30-positive cells. Lastly, the upregulation of WNT1 suggests enhanced Wnt signaling, which is often associated with increased cell proliferation and cancer progression, a finding supported by Huang et al. (2022) and Jin et al. (2020). Overall, these results indicate that Brentuximab selectively affects apoptotic and cell signaling pathways in cancer cells while having minimal impact on normal fibroblasts, consistent with its targeted therapeutic profile.

## Conclusion

The study on Brentuximab vedotin reveals promising findings, especially regarding its potential utility beyond traditional CD30-positive malignancies. The drug demonstrates significant efficacy in various cancers, including triple-negative breast cancer (MDA-MB-231) and ER-positive subtypes (MCF-7 and T-47D), suggesting that it may offer therapeutic benefits outside its established indications. Brentuximab’s impact on apoptotic markers like Caspase-3 and BAX confirms its role in inducing cell death through intrinsic apoptotic pathways, highlighting its broader applicability in oncology. However, the study has several limitations. The use of in vitro models, such as cancer cell lines, may not fully capture the complexities of human tumors in vivo. The observed effects in these models may not directly translate to clinical efficacy or safety. Furthermore, the study does not delve deeply into the potential off-target effects of Brentuximab or its underlying mechanisms in non-CD30-expressing cancers. The modest overall objective response rate (ORR) of 11% for CD30-positive solid tumors in previous studies indicates a need for optimization in treatment regimens and patient selection. Future research should involve animal models and clinical trials to validate these findings and evaluate the drug’s effectiveness and safety in a clinical context. Comprehensive studies addressing Brentuximab’s full spectrum of effects, including its impact on tumor microenvironments and patient populations, will be essential for advancing its use in cancer therapies.

## Data Availability

All data generated are presented in the current Manuscript.

## Abbreviations

ADCs: Antibody-drug conjugates
Annexin V-FITC: Annexin V labeled with fluorescein isothiocyanate
CD30: Cluster of differentiation 30
DMEM: Dulbecco’s Modified Eagle’s Medium with high glucose
DMSO: Dimethyl sulfoxide
DPBS: Dulbecco’s phosphate buffered saline
ER: Estrogen receptor
FBS: Fetal Bovine serum
FL: Fluorescence (detection channel in flow cytometry)
HSF: Human skin fibroblasts cell line
HL: Hodgkin lymphoma
MAbs: Monoclonal antibodies
MCF-7: Michigan cancer foundation-7 cell line
MDA-MB-231: M.D. Anderson - metastatic breast 231 cell line
MMAE: Monomethyl auristatin E
MTT: Methyl thiazol tetrazolium
NHL: Non-Hodgkin lymphoma
ORR: Objective response rate
PCR: Polymerase chain reaction
PI: Propidium iodide
qPCR: Quantitative real-time PCR
SEM: Standard error of the mean
T-47D: Human breast cancer cell line
TNBC: Triple-negative breast cancer
TNFRSF8: Tumor necrosis factor receptor superfamily member 8
U-937: Histiocytic lymphoma cell line
WHO: World Health Organization
